# Disparities in care and outcomes for primary liver cancer in England during 2008–2018: a cohort study of 8.52 million primary care population using the QResearch database

**DOI:** 10.1016/j.eclinm.2023.101969

**Published:** 2023-05-11

**Authors:** Weiqi Liao, Carol A.C. Coupland, Hamish Innes, Peter Jepsen, Philippa C. Matthews, Cori Campbell, The DeLIVER consortium, Eleanor Barnes, Emma Culver, Roman Fischer, Julia Hippisley-Cox, Hamish Innes, William L. Irving, Peter Jepsen, Matt Kelly, Paul Klenerman, Weiqi Liao, Derek Mann, Aileen Marshall, Philippa C. Matthews, Michael Pavlides, Rory J.R. Peters, Elisabeth Pickles, James Robineau, Benjamin Schuster-Böckler, Chunxiao Song, Jeremy Tomlinson, Christopher Welberry, Eleanor Barnes, Julia Hippisley-Cox

**Affiliations:** a Nuffield Department of Primary Care Health Sciences, University of Oxford, Oxford, UK; b Centre for Academic Primary Care, School of Medicine, University of Nottingham, Nottingham, UK; c School of Health and Life Sciences, Glasgow Caledonian University, Glasgow, UK; d Department of Hepatology and Gastroenterology, Aarhus University Hospital, Aarhus, Denmark; e The Francis Crick Institute, London, UK; f University College London, London, UK; g University College London Hospitals NHS Foundation Trust, London, UK; h Nuffield Department of Medicine, University of Oxford, Oxford, UK; i Oxford NIHR Biomedical Research Centre and Oxford University Hospitals NHS Foundation Trust, Oxford, UK

**Keywords:** Liver cancer, Hepatocellular carcinoma (HCC), Cholangiocarcinoma, Epidemiology, Disparities

## Abstract

**Background:**

Liver cancer has one of the fastest rising incidence and mortality rates among all cancers in the UK, but it receives little attention. This study aims to understand the disparities in epidemiology and clinical pathways of primary liver cancer and identify the gaps for early detection and diagnosis of liver cancer in England.

**Methods:**

This study used a dynamic English primary care cohort of 8.52 million individuals aged ≥25 years in the QResearch database during 2008–2018, followed up to June 2021. The crude and age-standardised incidence rates, and the observed survival duration were calculated by sex and three liver cancer subtypes, including hepatocellular carcinoma (HCC), intrahepatic cholangiocarcinoma (CCA), and other specified/unspecified primary liver cancer. Regression models were used to investigate factors associated with an incident diagnosis of liver cancer, emergency presentation, late stage at diagnosis, receiving treatments, and survival duration after diagnosis by subtype.

**Findings:**

7331 patients were diagnosed with primary liver cancer during follow-up. The age-standardised incidence rates increased over the study period, particularly for HCC in men (increased by 60%). Age, sex, socioeconomic deprivation, ethnicity, and geographical regions were all significantly associated with liver cancer incidence in the English primary care population. People aged ≥80 years were more likely to be diagnosed through emergency presentation and in late stages, less likely to receive treatments and had poorer survival than those aged <60 years. Men had a higher risk of being diagnosed with liver cancer than women, with a hazard ratio (HR) of 3.9 (95% confidence interval 3.6–4.2) for HCC, 1.2 (1.1–1.3) for CCA, and 1.7 (1.5–2.0) for other specified/unspecified liver cancer. Compared with white British, Asians and Black Africans were more likely to be diagnosed with HCC. Patients with higher socioeconomic deprivation were more likely to be diagnosed through the emergency route. Survival rates were poor overall. Patients diagnosed with HCC had better survival rates (14.5% at 10-year survival, 13.1%–16.0%) compared to CCA (4.4%, 3.4%–5.6%) and other specified/unspecified liver cancer (12.5%, 10.1%–15.2%). For 62.7% of patients with missing/unknown stage in liver cancer, their survival outcomes were between those diagnosed in Stages III and IV.

**Interpretation:**

This study provides an overview of the current epidemiology and the disparities in clinical pathways of primary liver cancer in England between 2008 and 2018. A complex public health approach is needed to tackle the rapid increase in incidence and the poor survival of liver cancer. Further studies are urgently needed to address the gaps in early detection and diagnosis of liver cancer in England.

**Funding:**

The *Early Detection of Hepatocellular Liver Cancer* (DeLIVER) project is funded by 10.13039/501100000289Cancer Research UK (Early Detection Programme Award, grant reference: C30358/A29725).

**Research in context**

**Evidence before this study**

Primary liver cancer is a global public health concern. Epidemiological studies of primary liver cancer in the UK have shown the incidence and mortality rates of liver cancer have increased substantially since 1980 and that men have a higher risk of being diagnosed with liver cancer than women. However, little is known about the disparities in care pathways and outcomes of primary liver cancer in the UK and the health inequality in stage at diagnosis, receiving treatments, and survival among people with different sociodemographic characteristics.

**Added value of this study**

This study provides up-to-date and comprehensive information on the liver cancer care pathways in England during 2008–2018 using a dynamic cohort of primary care population (8.52 million). The linked datasets from the QResearch database allow us to investigate the whole clinical pathways of liver cancer. The median observation period of patients diagnosed with liver cancer from entry into the database to the outcome (death or censored) was 11.1 years (minimum 1 year, maximum 31.3 years). We estimated survival for the three liver cancer subtypes for up to 13.4 years, which provides important statistics for liver cancer survival by stage in England. In addition, through investigating the clinical pathways of liver cancer, this study identifies the research gaps and provides a roadmap for future research in liver cancer in England.

**Implications of all the available evidence**

Around 40% of patients diagnosed with liver cancer were through emergency presentation. The proportion of patients diagnosed through the two-week wait referral pathway was only 11.7%. People in lower socioeconomic status were more likely to be diagnosed through emergency presentation. More efforts are needed to increase public awareness of liver cancer and the symptoms associated with liver cancer.

The risk of developing different liver cancer subtypes varies by ethnicity, socioeconomic deprivation, and geographical regions in England. This heterogeneity suggests a complex public health approach is needed to promote early detection and diagnosis of liver cancer in England that may need to tailor for different sub-populations and ethnic minority groups.

## Introduction

Liver cancer is a global public health concern. It was estimated that 905,700 people were diagnosed with and 830,200 people died from liver cancer globally in 2020 and the number of new cases and deaths from liver cancer could rise by 55% by 2040.^1^ Countries with high sociodemographic indexes had more pronounced increases.^2^ In the UK, the incidence and mortality rates for primary liver cancer increased by 257% and 239% from 1980 to 2013 respectively.^3^ In a study projecting cancer incidence and mortality in the UK until 2035, liver cancer is among the fastest increasing cancers in both incidence and mortality.^4^ According to the statistics by Cancer Research UK, the 1-year and 5-year relative survival estimates were around 38% and 13%, respectively.^5^ The rapid increase in incidence and mortality and poor survival of liver cancer is a huge burden to the NHS, patients, and carers. The UK government recognised the burden of liver diseases and liver cancer to society and published a guidance in 2015.^6^ Generally, patients diagnosed at earlier stages have better survival outcomes,^7^^,^^8^ as they have a greater chance of receiving treatments with curative intent. The two-week wait referral pathway, introduced by the Department of Health in 2000,^9^ is a rapid access strategy aiming to streamline referral for diagnosing cancer earlier and ultimately reducing cancer-related mortality. On the contrary, patients who received a cancer diagnosis after presenting to the accident and emergency department in the hospital (emergency presentation) have the poorest prognosis.^10^

Two previous studies have investigated the incidence, mortality, and survival of primary liver cancer in the UK during 1997–2017,^11^^,^^12^ providing research evidence to policymakers and healthcare commissioners to address geographical variations of liver cancer. However, little is known about health inequality in the care pathways and outcomes of primary liver cancer among people with different sociodemographic characteristics. Therefore, this study aims to provide an overview of the epidemiology of patients diagnosed with primary liver cancer in England in the recent decade (2008–2018, followed up until June 2021), to investigate the disparities in the whole liver cancer care pathway, to identify the gaps in current clinical practice, and to provide research evidence for policymakers to promote early detection and diagnosis of liver cancer and improve patient outcomes.

## Methods

### Study design, setting, population, and data sources

We used an open cohort of 8.52 million primary care population of individuals aged ≥25 years from 1335 general practices across England who were in the QResearch database (Version 46) between 1 January 2008 and 31 December 2018. However, some patients contributed data to the QResearch database preceding the start of the observation period (1 January 2008), some of whom could date back as early as 1989. The QResearch database is a Trusted Research Environment accredited by Health Data Research UK. It contains anonymised electronic health records (EHRs) from general practices using the Egton Medical Information Systems (EMIS). Patients’ primary care records are linked to Hospital Episode Statistics (HES, secondary care records, including inpatient, outpatient, accident and emergency), the cancer registry, and the death registry from the Office for National Statistics (ONS). At the point of data extraction for this study (August 2021), the most recent available data for liver cancer cases from cancer registry was 31 December 2018. Data for treatments and death outcomes were more contemporary (primary care data until 31 March 2021, HES until 31 May 2021, and ONS until 30 June 2021).

During data cleaning, we checked duplicated records to make sure there is no duplication in our cohort. Each patient who uses NHS services has a unique NHS number. Even if patients moved general practice, as long as the practices contribute data to the QResearch database, their records are linked through their unique NHS numbers, which were pseudonymised for research purposes to meet ethical requirements such as anonymity. However, patients may move to practices that are not contributing to the QResearch database, or patients may withdraw their consent to use their data for research purposes. In these two cases, patients were censored in the analysis at the point when they left the practice or withdrew consent.

### Definitions of primary liver cancer cases

**Fig. 1:**
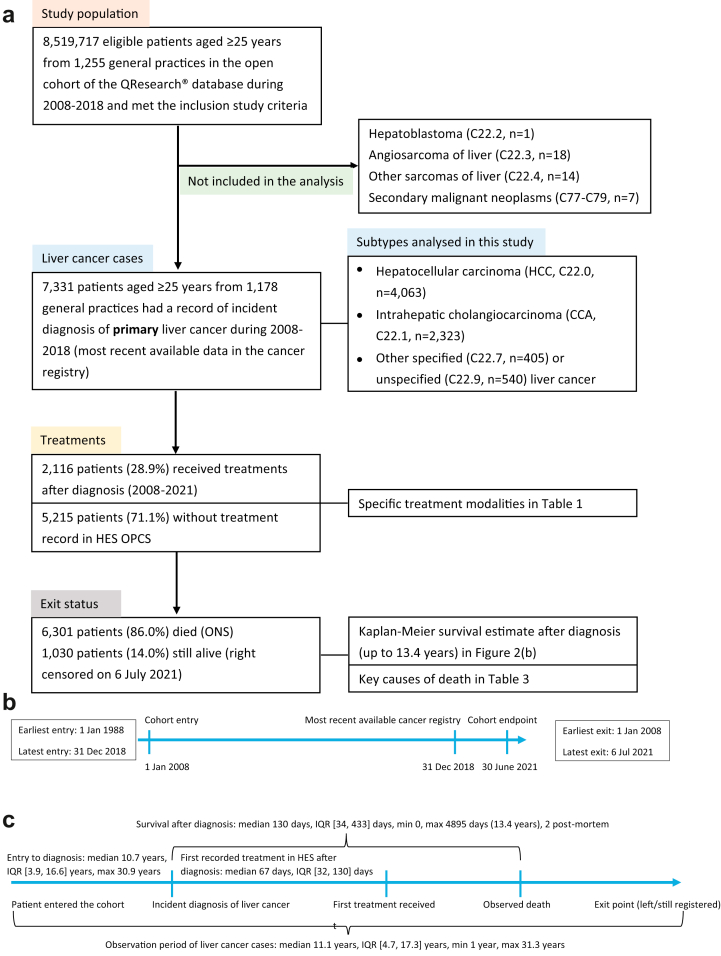
**Flowcharts and the timeline of the dynamic cohort of the English primary care population and the inclusion of primary liver cancer cases from the QResearch database (2008–2018, follow-up till June 2021).** (a) Flowchart of the dynamic cohort and the inclusion of primary liver cancer cases. (b) Timeline of the entry and exit date of the dynamic cohort of primary care population in the DeLIVER-QResearch study. (c) Intervals between the milestone events in the liver cancer care pathway. Note: Data extraction from the QResearch database (Version 46) was conducted in August 2021. The latest available data at the point of extraction in the four linked data sources were primary care records up to 31 March 2021, hospital episode statistics (HES) data up to 31 May 2021, Cancer Registration till 31 December 2018, and Office for National Statistics (ONS) mortality data till 30 June 2021.

The International Classification of Diseases (ICD-10) and for Oncology (ICD-O-2) codes were used to identify primary liver cancer cases (C22). Patients with additional ICD-10 codes (C77–C79, indicating secondary malignant neoplasm) were excluded to ensure that all the included cases were primary liver cancer. There were only a small proportion (<1%) of cases of hepatoblastoma (C22.2), angiosarcoma of liver (C22.3), and other sarcomas of liver (C22.4), also difficult to group them with other liver cancer subtypes. Therefore, we did not include them in the analysis. We grouped “other specified carcinomas of liver” (C22.7) and “liver, unspecified” (C22.9) together, and thus have three primary liver cancer subtypes in this study, which are hepatocellular carcinoma (HCC, C22.0 in ICD-10 code, or 817/818 in ICD-O-2 codes), intrahepatic cholangiocarcinoma (CCA, C22.1), and other specified or unspecified liver cancer (C22.7 and C22.9). The case definitions in this study are similar to previous studies.^13^ We provide a flowchart visualising the primary care population and how to select primary liver cancer cases (Fig. 1).

### Milestone events and outcomes in the liver cancer care pathway

We considered cancer diagnosis, receiving treatment, and death as milestone events in the clinical pathways of liver cancer. We analysed the incidence, routes to diagnosis,^10^ stage at diagnosis (from cancer registry), treatments for liver cancer (from HES), survival duration, and the most common causes of death (from ONS). Cancer staging was based on the TNM Classification of Malignant Tumours System (Stages I to IV, with missing/unknown stage), and cancer histology based on the ICD-10-O-2 system. Liver transplant, liver resection, and liver ablation were considered curative treatments in this study. Transarterial chemoembolisation (TACE) and selective internal radiation therapy (SIRT) are additional therapies for liver cancer included in the analysis. OPCS-4 Classification of Interventions and Procedures codes^14^ were used to identify these five procedures in the HES admission datasets. We also searched the OPCS codes in the HES outpatient dataset but no records were returned. Up to 15 causes of death (indexed by ICD-10 codes) were extracted from the ONS records. We reported the main causes of death by liver cancer subtypes.

### Definitions for calculating incidence, treatment interval, and survival duration

We calculated incidence rates, treatment intervals, and survival durations for the three liver cancer subtypes. The primary care population in the QResearch database is a dynamic cohort. People may enter or exit the cohort at any point as they register and de-register with participating general practices. When calculating the incidence using the study population, the start date was the latest of the study start date (1 January 2008, for those who were registered with participating practices before this date) or the entry date when patients entered the cohort after 1 January 2008 (either when they registered with participating practices or the date they turned 25 years old). The endpoint date was one of three situations, whichever came first: (1) the date of liver cancer diagnosis, (2) the date of death other than liver cancer (for the primary care population), or (3) the study end date (31 December 2018). Treatment interval was calculated between the date of cancer diagnosis and the earliest date of curative or any treatments recorded in the HES dataset for those who received treatments. Survival duration was from the date of liver cancer diagnosis to the date of death registered on the ONS (right censored on 30 June 2021).

### Statistical analysis

The research protocol and statistical analysis plan for the DeLIVER-QResearch project is peer-reviewed and published.^15^ Descriptive statistics were used to characterise the demographic and clinical features of the study cohort and patients diagnosed with three subtypes of liver cancer during follow-up. The crude and European age-standardised^16^ incidence rates for the three liver cancer subtypes were calculated and plotted by sex, calendar year, and liver cancer subtypes.

Cox regression was used to investigate whether patients' sociodemographic characteristics (i.e. age groups, sex, ethnicity, socioeconomic deprivation using Townsend quintile as a proxy) and the ten geographical regions in England were associated with an incident diagnosis of the three liver cancer subtypes. Individual socioeconomic status is not available in EHR, as this is sensitive and potentially identifiable personal information with ethical concerns. It is common practice to use an area-based index through ONS geocodes as a proxy for an individual's socioeconomic deprivation in UK health research. The QResearch database provides the Townsend score quintile, which is a measure of material deprivation including four domains (unemployment, non-car ownership, non-home ownership, and household overcrowding).^17^ Logistic regression was used to investigate the factors associated with (1) emergency presentation as route to cancer diagnosis compared with other routes and (2) late stage at diagnosis (Stages III or IV) compared with early stages (I or II), where patients with unknown stage were not included in the analysis. Competing risk regression (the Fine–Gray model)^18^ was used to investigate factors associated with receiving curative treatments or any of five treatments after diagnosis, with death as a competing risk for being unable to receive treatments. The Kaplan–Meier method was used to estimate the survival function for all-cause mortality and plot the survival curves by stage at diagnosis and liver cancer subtypes. Cox regression was used to investigate factors associated with survival duration, accounting for immortal time bias by considering the earliest treatment date.^19^^,^^20^ All the regression analyses were conducted by liver cancer subtypes. Interaction terms were explored when the main terms were statistically significant. A specific category “unknown” was used for missing data in all categorical variables. The statistical significance level was set as 0.05 in this study. Data were managed and analysed using Stata 17.0. The STrengthening the Reporting of OBservational studies in Epidemiology (STROBE) statement was used to guide the reporting of this study.

### Ethical approval

This study was approved by the QResearch Scientific Committee on 8 July 2021. QResearch is a Research Ethics Approved Database, confirmed by the East Midlands – Derby Research Ethics Committee (Research ethics reference: 18/EM/0400, project reference: OX30 DeLIVER). A dedicated webpage for this project is available on the QResearch website https://www.qresearch.org/research/approved-research-programs-and-projects/development-and-validation-of-personalised-risk-prediction-models-for-early-detection-and-diagnosis-of-hepatocellular-carcinoma-hcc-among-the-english-population-from-primary-care. The lay summary for this study, research protocol and statistical analysis plan are available from this webpage.

### Role of the funding source

The funders of the study had no role in study design, data collection, statistical analysis, interpretation of findings, writing this paper, or decision to publish.

## Results

### Descriptive epidemiology

#### Characteristics of the primary care cohort and the liver cancer cases

**Fig. 2:**
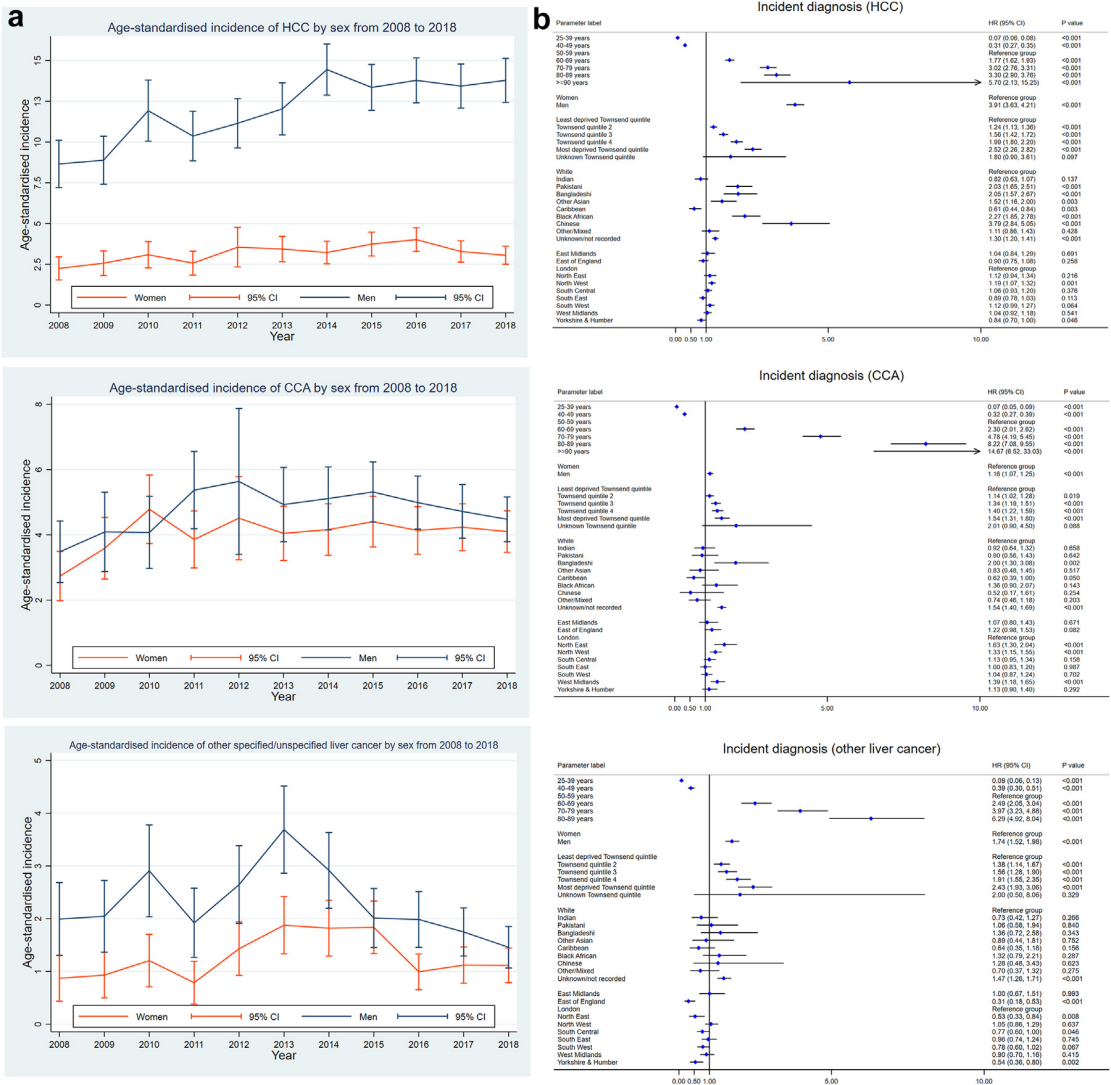
**Age-standardised incidence and factors significantly associated with an incident diagnosis of three liver cancer subtypes in the primary care population**. (a) Age-standardised incidence of three liver cancer subtypes, per 100,000 person-years in all three figures. (b) Factors significantly associated with an incident diagnosis of three liver cancer subtypes in the English primary care population (Cox regression). The figures show adjusted hazard ratios with 95% confidence interval (CI). HCC = hepatocellular carcinoma, CCA = cholangiocarcinoma.

Among the 8,519,717 individuals in the dynamic primary care cohort with 51,581,631 person-years of follow-up, 7331 patients were diagnosed with liver cancer, including 4063 HCC cases, 2323 CCA cases, and 945 cases of other specified/unspecified liver cancer. The sociodemographic characteristics of the primary care cohort and the liver cancer cases are shown in Table 1. The mean age for patients diagnosed with liver cancer was 69.9 years (standard deviation 12.0 years). Women were diagnosed at an older age than men in all three liver cancer subtypes. The sex distribution differed among the three liver cancer subtypes. The proportion of men in patients diagnosed with HCC was 77.9%, CCA 49.8%, and other specified/unspecified liver cancer 60.5%. The clinical characteristics of the three liver cancer subtypes are shown in Table 2, Table 3, Fig. 1, Fig. 2. The median observation period of patients diagnosed with liver cancer from entering the QResearch database to the endpoint (death or censoring) in this study was 11.1 years, interquartile range (IQR) [4.7, 17.3] years, minimum 1 year, while the longest observation was 31.3 years.

**Table 1:** Demographic characteristics of primary liver cancer cases and the cohort of primary care population (2008–2018) in the QResearch database at baseline.

	Population without liver cancer		Hepatocellular carcinoma		Cholangiocarcinoma		Other liver cancer		Total	
N	8,512,386	99.91%	4063	0.05%	2323	0.03%	945	0.01%	8,519,717	100.0%
Sex										
Female	4,199,271	49.3%	898	22.1%	1165	50.2%	373	39.5%	4,201,707	49.3%
Male	4,313,115	50.7%	3165	77.9%	1158	49.8%	572	60.5%	4,318,010	50.7%
Age at cohort entry (mean and SD)	48.8	15.5	63.3	11.8	67.2	11.8	65.1	12.1	48.9	15.6
Age bands at cohort entry										
25–39 years	1,363,697	16.0%	48	1.2%	14	0.6%	7	0.7%	1,363,766	16.0%
40–49 years	1,917,074	22.5%	164	4.0%	66	2.8%	44	4.7%	1,917,348	22.5%
50–59 years	1,782,738	20.9%	547	13.5%	214	9.2%	89	9.4%	1,783,588	20.9%
60–69 years	1,402,411	16.5%	1067	26.3%	475	20.4%	242	25.6%	1,404,195	16.5%
70–79 years	1,128,830	13.3%	1308	32.2%	729	31.4%	292	30.9%	1,131,159	13.3%
80–89 years	750,348	8.8%	846	20.8%	745	32.1%	246	26.0%	752,185	8.8%
90+ years	167,288	2.0%	83	2.0%	80	3.4%	25	2.6%	167,476	2.0%
Age at diagnosis by sex (mean and SD)										
Female			69.9	12.5	73.3	11.7	71.9	12.4		
Male			67.9	11.6	72.0	11.5	68.8	12.0		
Region in England										
East Midlands	197,580	2.3%	102	2.5%	56	2.4%	28	3.0%	197,766	2.3%
East of England	348,320	4.1%	148	3.6%	114	4.9%	15	1.6%	348,597	4.1%
London	2,226,126	26.2%	872	21.5%	333	14.3%	208	22.0%	2,227,539	26.1%
North East	211,216	2.5%	150	3.7%	106	4.6%	20	2.1%	211,492	2.5%
North West	1,551,246	18.2%	919	22.6%	543	23.4%	233	24.7%	1,552,941	18.2%
South Central	1,101,180	12.9%	495	12.2%	297	12.8%	105	11.1%	1,102,077	12.9%
South East	861,682	10.1%	334	8.2%	218	9.4%	108	11.4%	862,342	10.1%
South West	834,682	9.8%	474	11.7%	249	10.7%	98	10.4%	835,503	9.8%
West Midlands	835,706	9.8%	408	10.0%	291	12.5%	100	10.6%	836,505	9.8%
Yorkshire & Humber	344,648	4.0%	161	4.0%	116	5.0%	30	3.2%	344,955	4.0%
Townsend quintile										
1. Least deprived quintile	2,141,617	25.2%	937	23.1%	665	28.6%	217	23.0%	2,143,436	25.2%
2	1,844,672	21.7%	849	20.9%	558	24.0%	219	23.2%	1,846,298	21.7%
3	1,639,034	19.3%	787	19.4%	477	20.5%	182	19.3%	1,640,480	19.3%
4	1,471,542	17.3%	750	18.5%	354	15.2%	165	17.5%	1,472,811	17.3%
5. Most deprived quintile	1,393,292	16.4%	732	18.0%	263	11.3%	160	16.9%	1,394,447	16.4%
Not recorded	22,229	0.3%	8	0.2%	6	0.3%	≤5	0.2%	22,245	0.3%
Self-assigned ethnicity in GP record										
White	5,443,756	64.0%	2683	66.0%	1586	68.3%	635	67.2%	5,448,660	64.0%
Indian	230,236	2.7%	58	1.4%	31	1.3%	13	1.4%	230,338	2.7%
Pakistani	136,887	1.6%	91	2.2%	18	0.8%	11	1.2%	137,007	1.6%
Bangladeshi	91,332	1.1%	60	1.5%	22	0.9%	10	1.1%	91,424	1.1%
Other Asian	147,476	1.7%	54	1.3%	13	0.6%	8	0.8%	147,551	1.7%
Caribbean	104,632	1.2%	39	1.0%	18	0.8%	11	1.2%	104,700	1.2%
Black African	198,444	2.3%	109	2.7%	24	1.0%	16	1.7%	198,593	2.3%
Chinese	58,855	0.7%	48	1.2%	≤5	0.1%	≤5	0.4%	58,910	0.7%
Other	257,740	3.0%	63	1.6%	18	0.8%	10	1.1%	257,831	3.0%
Not recorded	1,843,028	21.7%	858	21.1%	590	25.4%	227	24.0%	1,844,703	21.7%

Note: In line with UK information governance recommendations, cells with counts ≤5 need to be suppressed in the tables to protect patient confidentiality.

SD = standard deviation.

**Table 2:** Clinical characteristics of the three subtypes of primary liver cancer (2008–2018): milestone events in the liver cancer care pathway.

	Hepatocellular carcinoma		Cholangiocarcinoma		Other liver cancer		Total	
Route to diagnosis (2008–2016)								
Death Certificate Only	11	0.4%	6	0.4%	6	0.8%	23	0.4%
Emergency presentation	999	34.1%	823	48.8%	322	43.1%	2144	40.0%
GP referral	917	31.3%	330	19.5%	190	25.4%	1437	26.8%
Inpatient elective	35	1.2%	38	2.3%	15	2.0%	88	1.6%
Outpatient	516	17.6%	186	11.0%	104	13.9%	806	15.0%
Two-Week Wait (TWW)	303	10.4%	248	14.7%	76	10.2%	627	11.7%
Unknown/Missing	146	5.0%	57	3.4%	34	4.6%	237	4.4%
Stage at diagnosis^a^								
Stage I (Earliest)	373	9.2%	47	2.0%	19	2.0%	439	6.0%
Stage II	404	9.9%	72	3.1%	22	2.3%	498	6.8%
Stage III	300	7.4%	92	4.0%	21	2.2%	413	5.6%
Stage IV (Latest)	497	12.2%	741	31.9%	145	15.3%	1383	18.9%
Unknown stage	2489	61.3%	1371	59.0%	738	78.1%	4598	62.7%
Liver transplant								
No	3928	96.7%	2321	99.9%	938	99.3%	7187	98.0%
Yes	135	3.3%	≤5	0.1%	7	0.7%	144	2.0%
Liver resection								
No	3831	94.3%	2221	95.6%	915	96.8%	6967	95.0%
Yes	232	5.7%	102	4.4%	30	3.2%	364	5.0%
Liver ablation								
No	3704	91.2%	2299	99.0%	907	96.0%	6910	94.3%
Yes	359	8.8%	24	1.0%	38	4.0%	421	5.7%
Patients received curative treatments^b^								
No	3386	83.3%	2199	94.7%	877	92.8%	6462	88.1%
Yes	677	16.7%	124	5.3%	68	7.2%	869	11.9%
Interval from diagnosis to first curative treatment (median days, IQR)	108	(35, 248)	54	(0, 141)	72	(29.5, 159.5)	93	(23, 230)
Transarterial chemoembolisation (TACE)								
No	3246	79.9%	2304	99.2%	883	93.4%	6433	87.8%
Yes	817	20.1%	19	0.8%	62	6.6%	898	12.2%
Selective internal radiation therapy (SIRT)								
No	4039	99.4%	2308	99.4%	941	99.6%	7288	99.4%
Yes	24	0.6%	15	0.6%	≤5	0.4%	43	0.6%
Patients received any of the above five treatments								
No	2710	66.7%	1791	77.1%	714	75.6%	5215	71.1%
Yes	1353	33.3%	532	22.9%	231	24.4%	2116	28.9%
Interval from diagnosis to any treatment (median days, IQR)	72	(36, 139)	56	(30, 103.5)	57	(30, 120)	67	(32, 130.5)
Patient's exit status								
Died (ONS record)^c^	3309	81.4%	2178	93.8%	814	86.1%	6301	86.0%
Left	229	5.6%	38	1.6%	45	4.8%	312	4.3%
Still registered with GP	525	12.9%	107	4.6%	86	9.1%	718	9.8%

Observed survival (95% CI)	Hepatocellular carcinoma	Cholangiocarcinoma	Other liver cancer
1-year (Female)	45.6% (42.3, 48.9)	25.5% (23.0, 28.1)	35.7% (30.7, 40.7)
1-year (Male)	49.1% (47.3, 50.8)	29.4% (26.8, 32.1)	43.0% (38.7, 47.2)
5-year (Female)	21.3% (18.6, 24.2)	5.0% (3.8, 6.5)	16.0% (12.3, 20.2)
5-year (Male)	21.0% (19.5, 22.5)	8.6% (7.0, 10.4)	19.3% (16.0, 22.9)
10-year (Female)	15.9% (13.1, 19.0)	3.5% (2.3, 5.1)	12.4% (8.9, 16.6)
10-year (Male)	14.1% (12.4, 15.8)	5.5% (4.0, 7.3)	12.9% (9.8, 16.4)

Notes: In line with UK information governance recommendations, cells with counts ≤5 need to be suppressed in the tables to protect patient confidentiality.

a Stage is based on the TNM staging system, where stage 1 is the earliest and stage 4 is the latest.

b Curative treatments included liver transplant, liver resection, and liver ablation.

c Death records were from 22 Jan 2008 to 30 June 2021.

**Table 3:** The top 10 causes of death (ICD-10 codes) for the three liver cancer subtypes (2008–2021).

HCC	ICD 10	Disease	Freq.	%
1	C220	Malignant neoplasm: Liver cell carcinoma	2766	83.6%
2	K746	Other and unspecified cirrhosis of liver	631	19.1%
3	E119	Type 2 diabetes mellitus: Without complications	297	9.0%
4	B182	Chronic viral hepatitis C	295	8.9%
5	I259	Chronic ischaemic heart disease, unspecified	206	6.2%
6	K729	Hepatic failure, unspecified	197	6.0%
7	I10	Essential (primary) hypertension	191	5.8%
8	C229	Malignant neoplasm: Liver, unspecified	175	5.3%
9	J449	Chronic obstructive pulmonary disease, unspecified	143	4.3%
10	J189	Pneumonia, unspecified	130	3.9%
**CCA**	**ICD 10**	**Disease**	**Freq.**	**%**

1	C221	Malignant neoplasm: Intrahepatic bile duct carcinoma	1981	91.0%
2	K830	Cholangitis	126	5.8%
3	I259	Chronic ischaemic heart disease, unspecified	105	4.8%
4	I10	Essential (primary) hypertension	101	4.6%
5	C80	Malignant neoplasm, without specification of site	98	4.5%
6	C798	Secondary malignant neoplasm of other specified sites	95	4.4%
7	C787	Secondary malignant neoplasm of liver and intrahepatic bile duct	81	3.7%
8	E119	Type 2 diabetes mellitus: Without complications	81	3.7%
9	J449	Chronic obstructive pulmonary disease, unspecified	75	3.4%
10	A419	Sepsis, unspecified	69	3.2%
**Other**	**ICD 10**	**Disease**	**Freq.**	**%**

1	C221	Malignant neoplasm: Intrahepatic bile duct carcinoma	223	27.4%
2	C229	Malignant neoplasm: Liver, unspecified	176	21.6%
3	C220	Malignant neoplasm: Liver cell carcinoma	122	15.0%
4	C80	Malignant neoplasm, without specification of site	109	13.4%
5	K746	Other and unspecified cirrhosis of liver	60	7.4%
6	C798	Secondary malignant neoplasm of other specified sites	49	6.0%
7	C259	Secondary malignant neoplasm of liver and intrahepatic bile duct	41	5.0%
8	C787	Malignant neoplasm: Pancreas, unspecified	41	5.0%
9	I259	Chronic ischaemic heart disease, unspecified	41	5.0%
10	E119	Type 2 diabetes mellitus: Without complications	39	4.8%

Note: HCC = hepatocellular carcinoma, CCA = cholangiocarcinoma, other = other specified/unspecified liver cancer.

The median number of causes of death was two for the three subtypes, IQR [1, 4] for HCC, [1, 3] for CCA and other specified/unspecified liver cancer. The maximum number of causes of death was 11 for HCC, 10 for CCA and other specified/unspecified liver cancer.

#### Incidence of liver cancer by subtype

The trends in age-standardised incidence rates of three liver cancer subtypes from 2008 to 2018 by sex are presented in Fig. 2(a). Generally, the age-standardised incidence rates increased over time, except for other specified/unspecified liver cancer in men. Notably, the age-standardised incidence rate of HCC increased dramatically in men over time by around 60%, from 8.7 (95% confidence interval, CI, 7.2–10.1) per 100,000 person-years in 2008 to 13.8 (12.4–15.1) per 100,000 in 2018.

#### *Diagnosis of liver cancer*

**Fig. 3:**
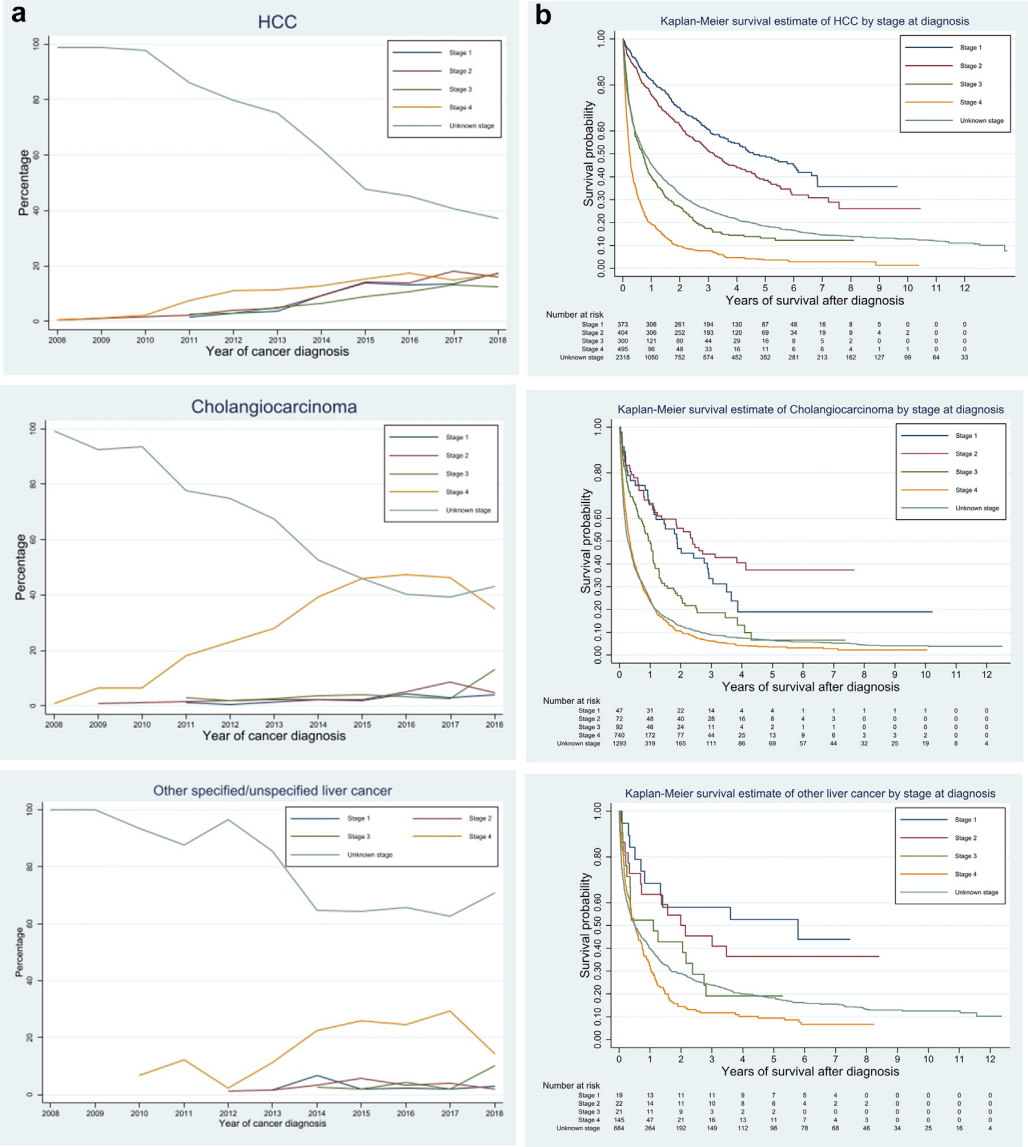
**Distribution of stage at diagnosis by calendar year and Kaplan–Meier survival curves by stage for the three sub-types of primary liver cancer**. (a) Stage at diagnosis. (b) Survival by stage. Note: HCC = hepatocellular carcinoma.

40% of patients diagnosed with liver cancer were through emergency presentation, which was the most common route to diagnosis, followed by GP referral (26.8%). The proportion of diagnoses via the two-week wait referral was only 11.7%. Fig. 3(a) shows the distribution of stage at diagnosis by year and liver cancer subtype. More than 60% of patients did not have stage information recorded (Table 2), although the proportion of unknown/missing stage decreased over time.

#### Receiving treatments

Among patients diagnosed with HCC, 16.7% received potentially curative treatments (liver transplant, resection, or ablation), while the proportion of patients diagnosed with CCA who received potentially curative treatments was only 5.3%. The median interval from diagnosis to receiving potentially curative treatments for liver cancer was 93 days, IQR [23–230] days. Among patients diagnosed with HCC, 33.3% received at least one treatment (potentially curative treatments, TACE, or SIRT), while the proportion for patients diagnosed with CCA was 22.9%, and for other specified/unspecified liver cancer was 24.4%. The median interval from diagnosis to receiving any of the five treatments was 67 days, IQR [32, 130] days.

#### Survival

86% of patients diagnosed with liver cancer died during the study observation period (Jan 2008–June 2021), with a median survival duration after diagnosis of 130 days, IQR [34, 433] days. The longest observed survival was 4895 days (13.4 years, right censored, Table 2 and Fig. 1(c)). Patients diagnosed with HCC lived longer than those diagnosed with CCA. Given that the overall survival of liver cancer is very poor and insufficient observation time for patients diagnosed after 2012, the numbers of patients surviving 10 years in each subtype were small. As shown in Fig. 3(b), the survival curve dropped with each incremental increase in stage. The survival curves for patients with unknown/missing cancer stage fell between those diagnosed in Stages III and IV.

#### Key causes of death in liver cancer cases

The top ten causes of death by liver cancer subtypes are summarised in Table 3. As expected, death from each liver cancer subtype was the main cause. Other key causes of death included liver cirrhosis (n = 718, 9.8% of all liver cancer cases, 718/7331), type 2 diabetes (n = 417, 5.7%), ischaemic heart disease (n = 352, 4.8%), and chronic hepatitis C (n = 315, 4.3%).

### Factors associated with incident liver cancer, emergency presentation, late stages at diagnosis, receiving treatments, and survival by liver cancer subtypes in regression models

#### Incident diagnosis of liver cancer

Age, sex, socioeconomic deprivation, ethnicity, and geographical region were all significant factors associated with an incident diagnosis of liver cancer by subtype in the primary care population (Fig. 2(b)). The hazard ratio (HR) for an incident diagnosis increased with escalating age and socioeconomic deprivation quintile in all three liver cancer subtypes. Compared with women, men were more likely to be diagnosed with liver cancer, but the HRs differed among subtypes, HR = 3.9, 95% CI (3.6–4.2) in HCC, 1.2 (1.1–1.3) in CCA, and 1.7 (1.5–2.0) in other specified/unspecified liver cancer. The risk of developing different liver cancer subtypes varied by ethnicity. Compared with white British, Chinese, Bangladeshi, Pakistani, other Asians, and Black Africans were more likely to be diagnosed with HCC (HR > 1), while Caribbeans were less likely (HR < 1). For CCA, the incidence was only higher in Bangladeshi, HR = 2.0 (1.3–3.1). In addition, people with unknown/missing ethnicity consistently had significantly increased risk (HR > 1) in all three liver cancer subtypes. We also observed geographical variation in the incidence of different liver cancer subtypes. Compared with London, Northwest England had a higher incidence rate for HCC and CCA, while Yorkshire & Humber had a lower incidence rate for HCC and other specified/unspecified liver cancer. Northeast and West Midlands had a higher incidence in CCA, while East of England, Northeast, and South Central had a lower incidence in other specified/unspecified liver cancer.

#### Emergency presentation

**Fig. 4:**
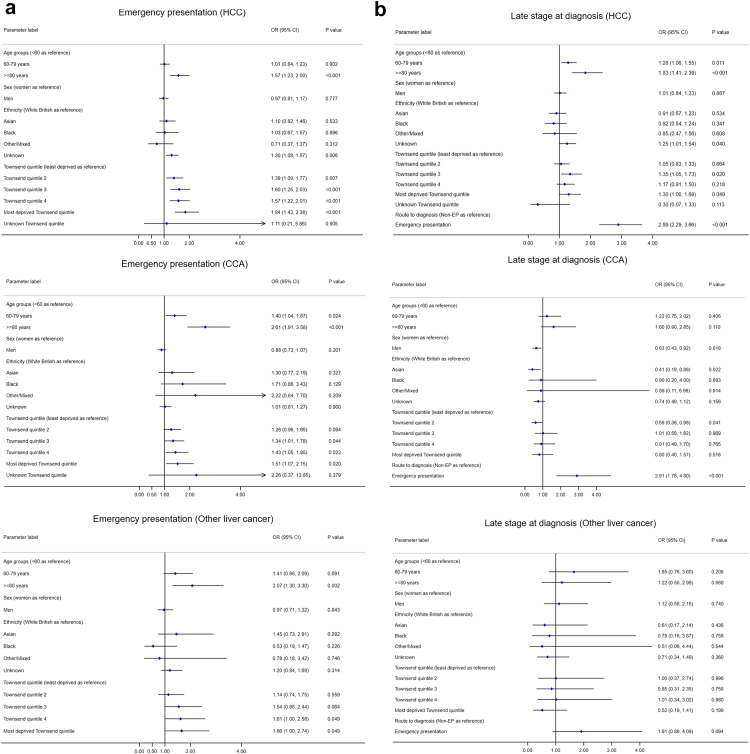

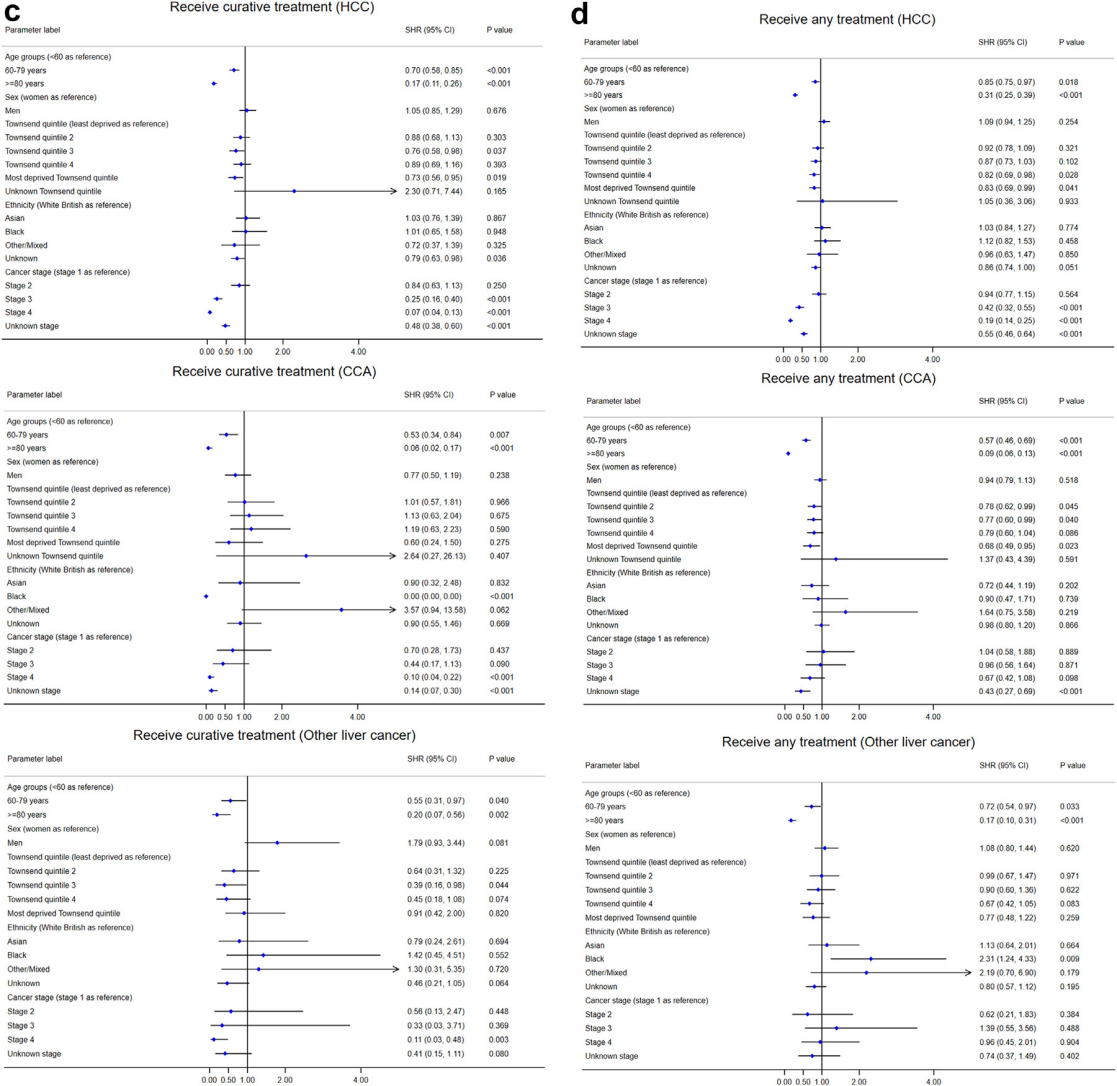

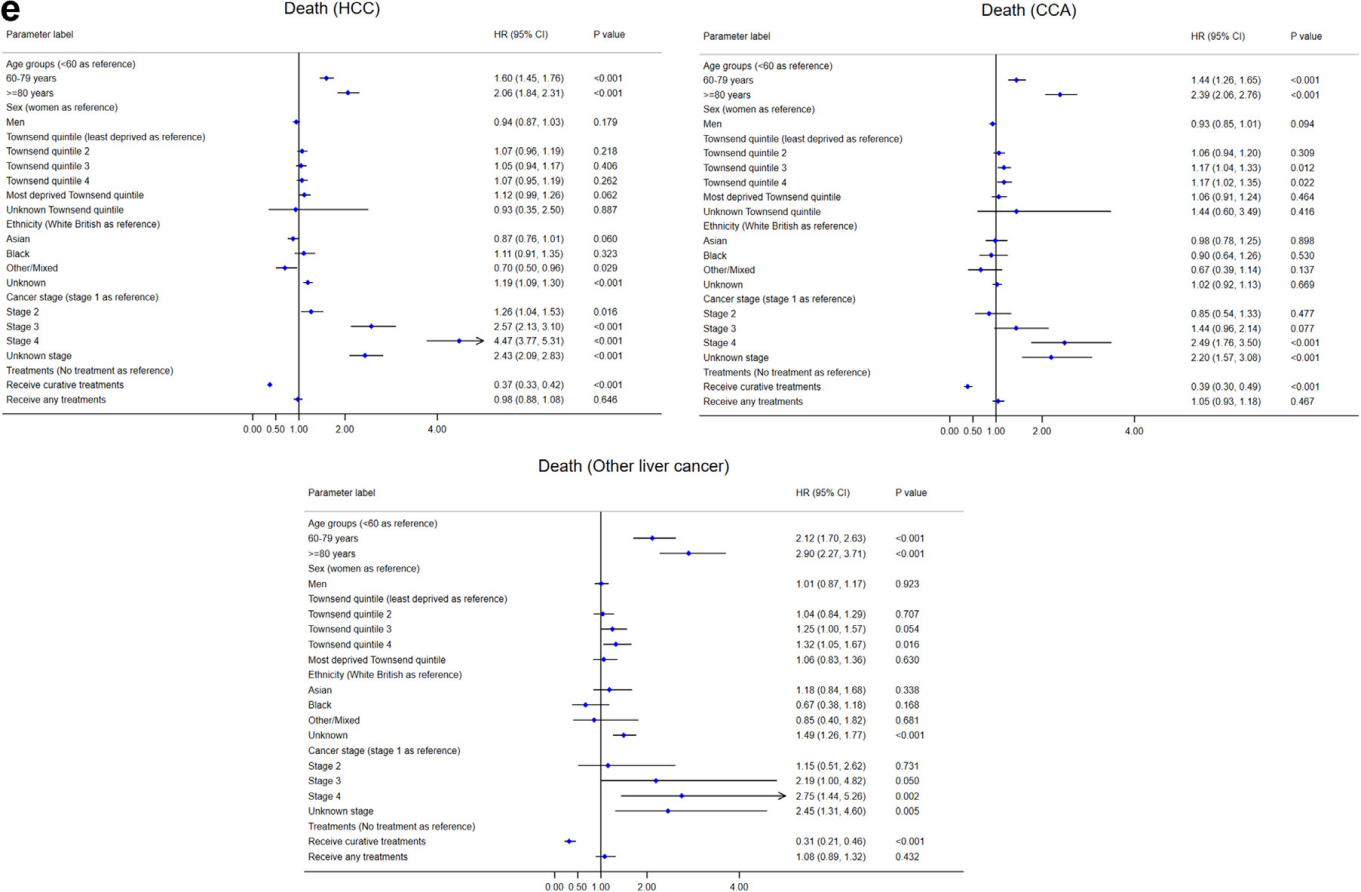
**Forest plots for the factors associated with emergency presentation, late stage at diagnosis, treatment, and survival after diagnosis for the three sub-types of primary liver cancer**. (a) Emergency presentation (Logistic regression, EP vs non-EP). (b) Late stage at diagnosis (Logistic regression, late vs early stages, early stages were defined as Stage I or II, while late stages were Stage III or IV). (c) Time to receive curative treatments (Competing risk regression). (d) Time to receive any treatment (Competing risk regression). (e) Survival duration (Cox regression). Note: HCC = hepatocellular carcinoma, CCA = cholangiocarcinoma.

Patients aged ≥80 years were significantly more likely to be diagnosed through an emergency presentation route in all three subtypes, compared to patients aged <60 years, with odds ratios (ORs) ranging from 1.6 (1.2–2.0) in HCC to 2.6 (1.9–3.6) in CCA (Fig. 4(a)). Socioeconomic deprivation was also significantly associated with emergency presentation, particularly in HCC. The ORs increased with each incremental quintile toward more deprivation. Sex was not significantly associated with emergency presentation.

#### Late stages at diagnosis

Compared with other routes to diagnosis, emergency presentation was significantly associated with diagnosis at late stages in HCC and CCA (both OR = 2.9, Fig. 4(b)). People of older age were more likely to be diagnosed in late stages in HCC. Males (compared with females, OR = 0.63) and Asians (compared with white British, OR = 0.41) were less likely to be diagnosed in late stages in CCA. No significant factors were identified for late stage diagnosis of other specified/unspecified liver cancer, probably due to the small number of people with known stages in this subtype (n = 207).

#### Receiving curative treatments or any treatments

Older patient groups and those with more advanced stages were less likely to receive curative treatments (liver surgery, transplant, or ablation) in all three subtypes in competing risk regression, where death was considered as a competing risk (Fig. 4(c)). Compared with patients in the least deprived quintile, patients in the third and most deprived quintiles were less likely to receive curative treatments for HCC. Compared with white British, people with unknown ethnicity was less likely to receive curative treatments for HCC, and Black Africans for CCA. Sex was not a significant factor.

Patients in older age groups were less likely to receive any treatments (the three curative treatments, TACE, and SIRT) in all three subtypes in competing risk regression (Fig. 4(d)). Patients diagnosed in advanced stages (III and IV) in HCC and unknown stage in HCC and CCA were less likely to receive any treatments. More deprived patients were less likely to receive any treatments for HCC and CCA. Compared with white British, patients with unknown/missing ethnicity were less likely to receive any treatments for HCC, and Black Africans for other specified/unspecified liver cancer. Sex was not significantly associated with receiving any treatments.

#### Survival duration after diagnosis

Older patients and those with advanced cancer stages were less likely to survive over a longer period in all three subtypes (Fig. 4(e)). Two interaction terms between age group and stage (≥80 years × Stage III and ≥80 years × Stage IV) were significant in survival in HCC, meaning that patients aged ≥80 years and diagnosed in advanced stages had poorer survival. The HR for survival in unknown cancer stage was close to Stage III in HCC, between Stages III and IV in CCA and other specified/unspecified liver cancer. Patients receiving curative treatment were more likely to survive longer (protective factor) in all three subtypes. Compared with white British, other/mixed ethnicity survived longer in HCC, while people with unknown ethnicity had lower survival in HCC and other specified/unspecified liver cancer. Compared with the least deprived quintile (Q1), patients in Q3 and Q4 in CCA and Q4 and unknown quintile in other specified/unspecified liver cancer survived shorter. Sex was not significantly associated with survival.

## Discussion

Among the 8.52 million English primary care population, 7331 patients were diagnosed with primary liver cancer. Heterogeneity was observed in the care pathways among three liver cancer subtypes. The age-standardised incidence rates have been increasing, particularly for HCC in men. Age, sex, socioeconomic deprivation, ethnicity, and geographical region were all significantly associated with an incident diagnosis of liver cancer at the population level. Older people were more likely to be diagnosed through emergency presentation and in late stages, less likely to receive treatments and survive. Patients with higher socioeconomic deprivation were more likely to be diagnosed through emergency route. Compared with white British, Asians and Black Africans were more likely to be diagnosed with HCC. This highlights a disproportionate disease burden in certain groups, and illustrates the potential for improvements in liver cancer outcomes if healthcare messages and resources are targeted appropriately at high-risk groups.

Considering the nature of this study is broadly descriptive analyses, we made some pragmatic decisions. The first one was how to operationalise age in different regression models. We used 10-year categories for age when we investigated factors associated with incidence, as the sample size for the whole primary care population was large. When we investigated factors associated with various liver cancer outcomes, we categorised age into three groups (<60, 60–79, and ≥80 years), but we did consider other possibilities. One was age as a continuous variable, and the other one was age in 10-year categories. If we used age as a continuous variable, we would get estimates of ORs or HRs for each incremental year of age. However, the risk of getting liver cancer and other outcomes among people in their 40s, 70s, or 80s was different. When we explored age in 10-year categories, the numbers of liver cancer cases in some strata (e.g. 25–29, 30–39, >90 years) and subtypes were too small and the models did not get accurate estimates for some age categories. In addition, the ORs/HRs were similar in some adjacent categories. Based on the initial exploratory analyses, we decided to use three age groups (<60, 60–79 and ≥ 80 years) for liver cancer cases, as the models converged and gave more precise estimates of the risks of age groups for different outcomes. But we will use fractional polynomials or restricted cubic splines to model the non-linear associations between continuous variables and the outcome when developing risk prediction models in our future studies.

Another pragmatic decision was that we used a specific category "unknown" for missing data in all categorical variables in this study. We have not made any assumptions about whether missing data were missing at random (MAR) or not, as we did not do any multiple imputation in this study. However, it makes sense to assume the missing ethnicity and Townsend score/quintile were missing at random, and the missing cancer stages were likely to be stage III or IV, as this was backed up by the survival curves and results from Cox regression.

The Barcelona Clinic Liver Cancer (BCLC) staging system was endorsed by the European Association for the Study of the Liver (EASL) to assess patients diagnosed with HCC, which was originally developed for stratification and treatment allocation.^21^ It incorporates tumour size and number, liver function, and patient performance status. However, BCLC is not available in the cancer registry. The generic TNM cancer staging system is used instead, which is based on pathological findings and does not include information on liver function and patient performance status for HCC. But the TNM system is also accepted by Cancer Research UK^22^ and the American Joint Committee on Cancer (AJCC).^21^ Although BCLC is useful to guide the management of HCC, the widely acknowledged TMN classification is suitable for our study, because TNM can be used in CCA and other specified/unspecified liver cancer, but not BCLC.

Men had a higher risk of getting diagnosed with the three liver cancer subtypes, but sex was not a significant factor associated with emergency presentation, receiving treatments, or survival. It may indicate there may be different aetiology and risk factors of developing liver cancer^23^ in different subtypes between men and women, such as differences in alcohol consumption, infection of hepatitis virus, occupational exposure, smoking, diet, BMI/obesity, or probably sex hormones.^24^ Given this, it may be worth developing and validating risk prediction models by sex to predict the future risk of liver cancer from asymptomatic population, which allows sex-specific risk stratification for early detection of liver cancer through screening.

Emergency presentation is known to be associated with worse outcomes (patients diagnosed at late stages and worse survival).^10^ 40% of patients were diagnosed through this route, and only 11.7% of patients were diagnosed through the two-week wait route. This may be because liver cancer is a less common cancer in the UK and the public is lack of awareness of this disease. Only one symptom (upper abdominal mass) was included in the NICE guideline [NG12]^25^ for recognition and referral of suspected liver cancer, which is a clear research gap in primary care cancer research. Together with a recent report that more symptomatic and larger tumours in North East and Cumbria,^26^ identifying a comprehensive list of symptoms significantly associated with liver cancer will be an important and urgent study. Both patients and GPs can benefit from this. Disseminating information about these symptoms to the general public can increase public awareness of symptoms related to liver cancer, which can help patients seek medical review from their GPs more promptly. It could also help GP better manage patients with symptomatic presentation and refer them for further investigation if necessary, which may increase the proportion of two-week referrals and decrease the proportion of emergency presentation.

Due to its poor survival, liver cancer was considered as “a cancer of unmet need” and added into the Cancer Research UK 2022 research strategy.^27^ The disparity in incidence in age groups, sex, ethnicity, socioeconomic deprivation, and geographical regions suggested that it is a complex public health issue to early detect and diagnose liver cancer in the UK. Different approaches may be needed to target different sub-populations and regions. It is important to increase public awareness of liver cancer in the UK, especially for people in higher socioeconomic deprivation and ethnic minorities (Asians and Africans). Socioeconomic deprivation was associated with increased incidence rates, emergency presentation, and being less likely to receive treatments, which is a gap to address to reduce health inequalities in liver cancer care.

Some studies^11^, ^12^, ^13^^,^^28^, ^29^, ^30^ have reported the incidence and mortality of liver cancer in the UK and have investigated the trend as early as the 1970s.^30^ We compared our findings with the two most recent papers published by Burton et al.^11^^,^^12^ The first paper focused on the incidence, incidence-based mortality, and survival of three subtypes of primary liver cancer during 2000–2015 by sex in the four UK nations.^11^ The second investigated the regional variations in the incidence, routes to diagnosis, treatments, and survival of HCC among 19 cancer alliances across England during 2010–2016.^12^ Most findings and trends were similar between our study and Burton's two studies. Burton et al. focused on reporting the temporal trends and geographical variations of liver cancer. While we have covered these two aspects in our study, we focused more on the population and patient characteristics (such as age groups, sex, socioeconomic deprivation, and ethnicity) of the three liver cancer subtypes, as our study aimed to provide evidence to improve our understanding of the disparities among sub-populations with different characteristics in the liver cancer care pathway and the gaps in current clinical practice. Furthermore, we have strengths in providing long-term survival estimates and investigating factors associated with several milestone events (route to diagnosis, stage, receiving treatment, and survival).

During the COVID-19 pandemic, healthcare resources have been diverted and prioritised for the COVID-19 disease, which influenced chronic disease management and disrupted routine health checks for adults aged 40–74 years in England. A recent study reported that the COVID-19 pandemic resulted in a 37% reduction in the number of incident HCC cases in North East and Cumbria.^26^ Fewer patients were detected by surveillance or as part of routine care, with an increase in symptomatic and larger tumours. We did not have the data to evaluate the impact of the COVID-19 pandemic on the early diagnosis of liver cancer in this study, as cancer registry data often have a time lag of three years in England. However, we can foresee it will be challenging for the NHS to tackle the backlog in the post-COVID era.

This study has three key strengths. Firstly, we used a representative cohort consisting of approximately 20% of the whole English primary care population. Primary care provides a unique opportunity to promote early cancer diagnosis due to its almost whole population coverage and free of charge at the point of care delivery in the UK. Study findings from the primary care population have the potential to inform health policy for population health and translate into clinical practice. Secondly, this paper reports up-to-date statistics of incidence and survival based on long periods of clinical follow-up, as well as comprehensive information on the milestone events in the clinical pathways of liver cancer in England, while most papers on cancer epidemiology only report the overall statistics of incidence and mortality by age groups and sex. We focused on ethnicity and socioeconomic deprivation in this study, which may help health commissioners and policymakers to consider health equality in different geographical regions and reduce health inequities in ethnic minority groups and people in lower socioeconomic status. Thirdly, this study also benefited from data linkage, which provided important information on cancer stage, histology, treatment modalities, and causes of death for primary liver cancer cases. In addition, the data allowed us to observe the whole population for a long period. We were able to report observed survival of liver cancer by sex and subtypes for up to 13 years, while the Cancer Research UK liver cancer survival statistics only have 1-year and 5-year survival on its website, but not 10-year survival statistics and survival by stage.^5^

As with many other observational studies, our study has limitations and potential biases. This study is focused on the three basic elements of epidemiology in liver cancer: temporal trends, geographical distribution, and population characteristics, i.e. time, place, and people. We did not investigate the aetiology (e.g. hepatitis B/C virus infection, alcohol, non-alcoholic fatty liver disease, etc.) of primary liver cancer, since this is not the primary aim of our study, but it is a limitation. We will explore how well these aetiologies are recorded in the UK primary care population in future studies. Another limitation is there may be some coding errors in the “other specified/unspecified liver cancer” group, which could result in misclassification bias of either the outcomes or the associated features, for example, inconsistencies between diagnoses in the cancer registration dataset and causes of death in the death registration dataset in this subgroup. Finally, individual socioeconomic status is not available in EHR, Townsend score quintile was used as a proxy for material deprivation^17^ instead. However, this is a common practice in UK health research.

We have conceptualised and planned studies to contribute to the early detection and diagnosis of liver cancer from the English primary care setting and at the population level in the next step. First, we aim to identify a comprehensive list of significant symptoms and comorbidities associated with liver cancer for early diagnosis through symptomatic presentation in primary care. This may inform the update of the current NICE guideline [NG12]^25^ to include more symptoms in the referral pathways for suspected liver cancer. Secondly, we will develop and validate risk prediction models to predict incident liver cancer. The risk prediction model can identify individuals at high risk from the asymptomatic population who may benefit from surveillance and screening for early detection of liver cancer. We will use the same English primary care cohort for these studies.

In conclusion, this study provides a comprehensive overview of the changing epidemiology and the disparities in the clinical pathways of primary liver cancer in England during 2008–2018. It is a complex public health issue to tackle the rapid growth of liver cancer incidence and the poor survival. Different strategies may be needed for different sub-populations and geographical regions. Further studies are urgently needed to address the gaps in early detection and diagnosis of liver cancer.

## Contributors

EB and JH-C secured the funding for this study. EB is the chief investigator of the DeLIVER programme, and JH-C is the package lead and the guarantor of this study. WL conducted the literature review, specified the data, and led the ethical approval. WL designed the statistical analysis plan, with methodological input from JH-C, CACC, PJ and HI, clinical and contextual input from PJ, JH-C, HI, EB, PM and CC. WL and JH-C have full access and verified the study data. WL performed statistical analyses and prepared the tables and figures. All authors contributed to the interpretation of the findings. WL drafted the whole manuscript. All authors read and commented on the earlier drafts, contributed to the revision of the manuscript, approved the final version of the manuscript, and had final responsibility for the decision to submit for publication.

## Data sharing statement

To guarantee the confidentiality of personal and health information of patients, only the named authors have had full access to the data during the study, in accordance with the relevant licence agreements. Information on access to the QResearch data is available on the QResearch website (www.qresearch.org).

## Declaration of interests

JH-C is an unpaid director of QResearch, a not-for-profit organisation in a partnership between the University of Oxford and EMIS Health, who supply the QResearch database for this work. JH-C is a founder and shareholder of ClinRisk Ltd and was its medical director until 31 May 2019. ClinRisk Ltd produces open and closed source software to implement clinical risk algorithms into clinical computer systems. EB contributed to patents on imaging technologies that are owned by Perspectum Diagnostics, an imaging spin-out company of the University of Oxford, and holds shares of Perspectum Diagnostics. EB received honoraria from Roche Diagnostics for a presentation at a symposium and for contributions/evaluations of a manuscript. PJ received research funding from Novo Nordisk Foundation as a Borregaard Clinical Ascending Investigator (grant reference number NNF19OC0054612). The funder had no role in this study. The University of Oxford received funding from GSK, which partly contributed to a DPhil studentship for CC in HBV and HCC epidemiology. The DeLIVER consortium has Roche, Perspectum Diagnostics, and Oncimmune as industry partners. WL, CACC, and HI have no interests to declare for this work.

## Appendix A Supplementary data

Deliver Consortium authorship

Checklist of the STROBE statement

## References

[1] Rumgay H., Arnold M., Ferlay J. (2022). Global burden of primary liver cancer in 2020 and predictions to 2040. J Hepatol.

[2] Liu Z., Jiang Y., Yuan H. (2019). The trends in incidence of primary liver cancer caused by specific etiologies: results from the Global Burden of Disease Study 2016 and implications for liver cancer prevention. J Hepatol.

[3] Oke J.L., O'Sullivan J.W., Perera R., Nicholson B.D. (2018). The mapping of cancer incidence and mortality trends in the UK from 1980-2013 reveals a potential for overdiagnosis. Sci Rep.

[4] Smittenaar C.R., Petersen K.A., Stewart K., Moitt N. (2016). Cancer incidence and mortality projections in the UK until 2035. Br J Cancer.

[5] Cancer Research UK (2022). Liver cancer statistics. https://www.cancerresearchuk.org/health-professional/cancer-statistics/statistics-by-cancer-type/liver-cancer.

[6] Office for Health Improvement and Disparities (the UK government) (2015).

[7] American Cancer Society (2022). Liver cancer survival rates. https://www.cancer.org/cancer/liver-cancer/detection-diagnosis-staging/survival-rates.html.

[8] Cancer Research UK (2018). Liver cancer survival. https://www.cancerresearchuk.org/about-cancer/liver-cancer/survival.

[9] Department of Health, Health Do (2000). The NHS cancer plan.

[10] Elliss-Brookes L., McPhail S., Ives A. (2012). Routes to diagnosis for cancer - determining the patient journey using multiple routine data sets. Br J Cancer.

[11] Burton A., Tataru D., Driver R.J. (2021). Primary liver cancer in the UK: incidence, incidence-based mortality, and survival by subtype, sex, and nation. JHEP Rep.

[12] Burton A., Balachandrakumar V.K., Driver R.J. (2022). Regional variations in hepatocellular carcinoma incidence, routes to diagnosis, treatment and survival in England. Br J Cancer.

[13] Konfortion J., Coupland V.H., Kocher H.M., Allum W., Grocock M.J., Jack R.H. (2014). Time and deprivation trends in incidence of primary liver cancer subtypes in England. J Eval Clin Pract.

[14] NHS Digital (2021).

[15] Liao W., Jepsen P., Coupland C. (2022). Development and validation of personalised risk prediction models for early detection and diagnosis of primary liver cancer among the English primary care population using the QResearch database: research protocol and statistical analysis plan. Diagnos Prognos Res.

[16] European Union (2013).

[17] Townsend P., Davidson N. (1982).

[18] Fine J.P., Gray R.J. (1999). A proportional hazards model for the subdistribution of a competing risk. J Am Stat Assoc.

[19] Levesque L.E., Hanley J.A., Kezouh A., Suissa S. (2010). Problem of immortal time bias in cohort studies: example using statins for preventing progression of diabetes. BMJ.

[20] Yadav K., Lewis R.J. (2021). Immortal time bias in observational studies. JAMA.

[21] Brown Z.J., Tsilimigras D.I., Ruff S.M. (2023). Management of hepatocellular carcinoma: a review. JAMA Surg.

[22] Cancer Research Uk (2023). Liver cancer Stages (TNM staging). https://www.cancerresearchuk.org/about-cancer/liver-cancer/stages/tnm-staging.

[23] Chen C.L., Kuo M.J., Yen A.M. (2020). Gender difference in the association between metabolic factors and hepatocellular carcinoma. JNCI Cancer Spectr.

[24] Yeh S.H., Chen P.J. (2010). Gender disparity of hepatocellular carcinoma: the roles of sex hormones. Oncology.

[25] The National Institute for Health and Care Excellence (NICE) (2020). Suspected cancer: recognition and referral. https://www.nice.org.uk/guidance/ng12.

[26] Geh D., Watson R., Sen G. (2022). COVID-19 and liver cancer: lost patients and larger tumours. BMJ Open Gastroenterol.

[27] Cancer Research Uk (2022). Research opportunities in cancers of unmet need. https://www.cancerresearchuk.org/funding-for-researchers/research-opportunities-in-harder-to-treat-cancers.

[28] Coupland V.H., Kocher H.M., Berry D.P. (2012). Incidence and survival for hepatic, pancreatic and biliary cancers in England between 1998 and 2007. Cancer Epidemiol.

[29] Jack R.H., Konfortion J., Coupland V.H. (2013). Primary liver cancer incidence and survival in ethnic groups in England, 2001-2007. Cancer Epidemiol.

[30] Ladep N.G., Khan S.A., Crossey M.M., Thillainayagam A.V., Taylor-Robinson S.D., Toledano M.B. (2014). Incidence and mortality of primary liver cancer in England and Wales: changing patterns and ethnic variations. World J Gastroenterol.

